# Kongenitale periorbitale Hautveränderungen bei einem weiblichen Säugling – Was tun bei „raccoon eyes?“

**DOI:** 10.1007/s00105-025-05581-2

**Published:** 2025-09-23

**Authors:** Ara Cho, Babak Adib, Adrian Tanew, Sonja Radakovic

**Affiliations:** 1 Universitätsklinik für Dermatologie, Medizinische Universität Wien, Währinger Gürtel 18–20, 1090 Wien, Österreich; 2 Wien, Österreich

**Keywords:** Neonataler Lupus erythematodes, Periorbitales Erythem, Kongenitale Hautveränderung, Anti-SSA/Ro-Antikörper, Anti-SSB/La-Antikörper, Neonatal lupus erythematosus, Periorbital erythema, Congenital skin lesion, Anti-SSA/Ro antibodies, Anti-SSB/La antibodies

## Abstract

Ein 4 Monate altes weibliches Baby wurde wegen seit Geburt bestehender periorbitaler Hautveränderungen vorstellig. Der Säugling zeigte bilaterale periorbitale Erytheme, Teleangiektasien und ekzematöse Hautveränderungen. Aufgrund der typischen Klinik („raccoon eyes“) wurde die Verdachtsdiagnose eines neonatalen Lupus erythematodes (NLE) gestellt, die durch erhöhte Anti-Ro-Antikörper beim Kind und seiner Mutter bestätigt werden konnte. Kardiologische oder andere Anomalien wurden nicht festgestellt.

## Abstract (en)

A 4-month-old female infant presented with congenital periorbital skin changes. The patient showed bilateral periorbital erythema, telangiectasia and eczematous skin lesions resembling “raccoon eyes” which resulted in a clinical diagnosis of neonatal lupus erythematosus. The diagnosis was confirmed by the detection of elevated anti-Ro antibodies in both the infant and her mother. No cardiological or other abnormalities were found.

## Anamnese

Ein 4 Monate altes Mädchen wurde vom niedergelassenen Facharzt wegen kongenitaler periorbitaler Hautveränderungen zur Begutachtung in unsere Ambulanz überwiesen. Das Baby war das erstgeborene Kind anamnestisch gesunder Eltern, und sowohl Schwangerschaft als auch Geburt waren komplikationslos verlaufen. Die bisherige Entwicklung des Kindes war altersentsprechend, und neben den periorbitalen Hautveränderungen gab es keine weiteren Auffälligkeiten. Allerdings berichteten die Eltern, dass sich bei ihrem Kind nach einem 20-minütigen Spaziergang im Freien eine deutliche periorbitale Schwellung entwickelt habe, die sich innerhalb kurzer Zeit spontan rückgebildet habe (Abb. 1).

**Abb. 1:**
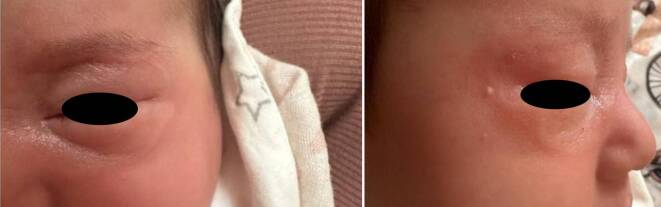
2. Lebensmonat; periorbitale Schwellung nach 20-minütigem Aufenthalt im Freien

Obwohl ursprünglich beide Elternteile Vorerkrankungen verneint hatten, wurden nach gezielter Befragung von der Kindesmutter eine diskrete Raynaud-Symptomatik und minimale Gelenksschmerzen bei Belastung angegeben. Bei beiden Elternteilen bestand anamnestisch kein Hinweis auf eine allergische Disposition oder Ekzemneigung, auch Routine-Laboruntersuchungen waren stets unauffällig gewesen. Allergologische Laborparameter waren bei fehlender Anamnese nicht bestimmt worden.

Aufgrund des klinischen Bildes wurde die Verdachtsdiagnose eines neonatalen Lupus erythematodes (NLE) gestellt. Differenzialdiagnostisch kamen primär ein periorbitales atopisches oder seborrhoisches Ekzem infrage, im weiteren Sinne auch eine hämatologische Grunderkrankung mit kutaner Manifestation.

## Befunde

**Abb. 2:**
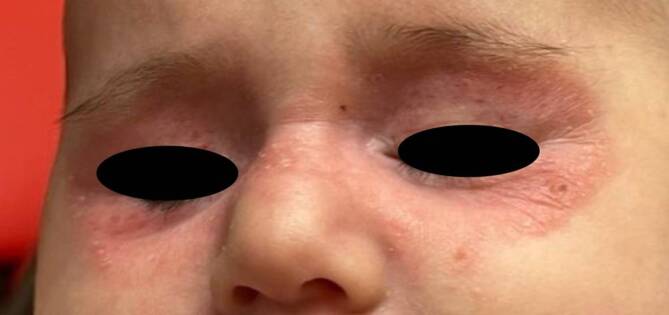
Erstvorstellung; 4. Lebensmonat; stark ausgeprägte bilaterale ekzematöse Hautveränderungen um die Periorbitalregion und Teleangiektasien im Bereich der Oberlider

Im dermatologischen Status des Kindes zeigte sich ein bilaterales periorbitales Erythem mit Teleangiektasien und mäßig ausgeprägtem Ekzem (Abb. 2). Das übrige Integument einschließlich der Schleimhäute und Hautanhangsgebilde war unauffällig.

Abgesehen von den Hautveränderungen ergab die pädiatrische Untersuchung den Befund eines gesunden Mädchens mit normaler, altersentsprechender Entwicklung. Die echokardiographische Untersuchung erbrachte keinen Hinweis auf das Vorliegen eines atrioventrikulären Blockes (AV-Block).

In der Immunserologie fanden sich bei der Patientin ein positiver Befund für antinukleäre Autoantikörper (Immunoassay 1.6 Ratio; positiv ab > 1.1), grenzwertig positive dsDNA-Antikörper (13 IU/ml; positiv ab 10 IU/ml) und positive anti-SSA/Ro Antikörper (> 240 U/ml). Alle weiteren laborchemische Befunde waren im Normbereich.

Die dermatologische Untersuchung der Kindesmutter erbrachte keinen pathologischen Befund. Eine wegen der Raynaud Symptomatik durchgeführte Kapillarmikroskopie war ebenso unauffällig. Immunserologisch nachgewiesen wurden auch bei der Kindesmutter erhöhte ANA (1:160 U/ml), Anti-SSA/Ro- (> 240 U/ml) und zusätzlich Anti-SSB/La-Antikörper (11.0 U/ml) nachgewiesen.

## Diagnose

Die Zusammenschau aller klinischen und laborchemischen Befunde des Kindes und dessen Mutter stand im Einklang mit der initialen klinischen Verdachtsdiagnose eines NLE. Hierbei handelt es sich um eine erworbene kongenitale Autoimmunerkrankung, welche durch die diaplazentare Übertragung maternaler Anti-SSA/Ro‑, Anti-SSB/La- oder Anti-U1-RNP-Antikörper auf den Feten während der Schwangerschaft ausgelöst wird [1]. Von einer zusätzlichen Diagnosesicherung mittels Gewebebiopsie wurde abgesehen, einerseits aufgrund des jungen Alters des Mädchens, andererseits da sich bereits bei der nächsten Kontrolle nach 2 Monaten die erwartete Besserung des Lokalbefundes abgezeichnet hatte.

## Therapie und Verlauf

Der NLE bedarf zumeist keiner topischen oder systemischen Therapie. Die meisten klinischen Manifestationen bilden sich üblicherweise mit dem Abbau der maternalen Antikörper spontan zurück.

Bei unserer Patientin wurden eine indifferente Pflegetherapie für den Augenbereich verschrieben und eine absolute Sonnenkarenz empfohlen. Bei den klinischen Nachkontrollen zeigten sich eine deutliche Rückbildung der periorbitalen Hautveränderungen (Abb. 3). Im 8. Lebensmonat war das Erythem komplett abgeblasst, lediglich diskrete Teleangiektasien waren noch vorhanden (Abb. 4). Die maternalen Anti-SSA/Ro-Antikörper waren serologisch nach dem 12. Lebensmonat nicht mehr nachweisbar. Der spontan remittierende Verlauf unterstützte die klinische Arbeitsdiagnose eines NLE und entsprach der zu erwartenden Abheilungstendenz eines NLE in zeitlicher Korrelation zum Abbau der maternalen Antikörper beim Feten. Die weitere Entwicklung des Mädchens verlief altersentsprechend und komplikationslos.

**Abb. 3:**
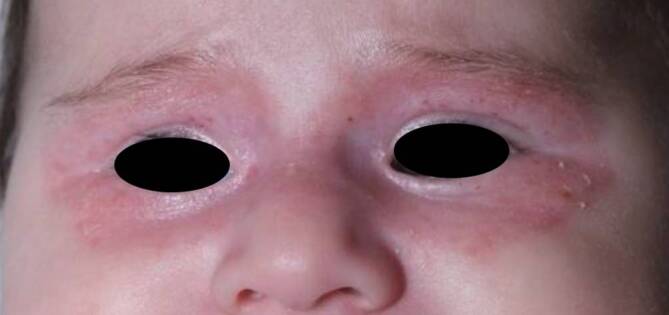
6. Lebensmonat; regredientes bilaterales Erythem nach Pflegetherapie und Sonnenkarenz

**Abb. 4:**
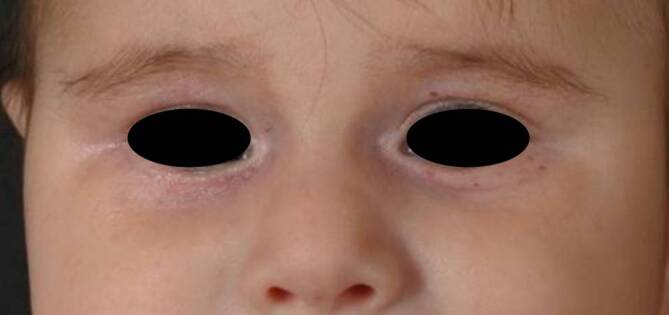
8. Lebensmonat; vollständige Regression des periorbitalen Hautveränderungen bis auf vereinzelte Teleangiektasien

## Diskussion

Bei kongenitalen periorbitalen Hautveränderungen, die an „raccoon eyes“ erinnern, muss primär an die Diagnose eines NLE gedacht und unverzüglich eine kardiologische Untersuchung zum Ausschluss einer angeborenen Herzrhythmusstörung eingeleitet werden. Bei negativer Familienanamnese sollte eine gezielte Befragung und Untersuchung der Kindesmutter erfolgen, um latente, vernachlässigte oder falsch interpretierte klinische Zeichen eines systemischen Lupus erythematodes auszuschließen [1]. Der Nachweis von Anti-SSA/Ro- und Anti-SSB/La-Antikörpern bei der Mutter sollte eine weiterführende Lupus-Diagnostik zur Folge haben [2, 3].

Kutane Manifestation im Rahmen eines NLE treten bei 5–16 % der Neugeborenen auf. Es handelt sich dabei um transiente Hautveränderungen, vor allem periorbital, selten auch am Körper. Das klinische Erscheinungsbild der „raccoon eyes“ ist ein wichtiger Hinweis für das Vorliegen eines NLE. Aufgrund der Seltenheit der Erkrankung werden die kutanen Veränderungen häufiger fehldiagnostiziert als ein gewöhnliches Ekzem oder eine mykotische Infektion, was zu einer Verzögerung der Diagnosestellung führen kann. Hautveränderungen im Rahmen eines NLE sind zumeist selbstlimitierend und zeigen in der Regel nach dem 6. bis 8. Lebensmonat eine spontane komplette Remission. Allerdings können in 10–25 % der Fälle kutane Manifestationen wie Teleangiektasien, Dyspigmentierung (sowohl Hyper- als auch Hypopigmentierung), Vernarbung oder Hautatrophie bis zum 4. Lebensjahr persistieren [4]. Die Wahrscheinlichkeit kutaner Manifestationen bei einem neugeborenen Kind ist deutlich höher, wenn bei der Mutter nicht nur Anti-SSA/Ro-, sondern auch Anti-SSB/La-Antikörper vorhanden sind [5].

Die Organmanifestationen im Rahmen eines NLE können vielfältig sein. Neben den am häufigsten auftretenden Hautveränderungen kann eine kardiale Mitbeteiligung in Form eines kongenitalen AV-Blockes bei etwa 2–5 % der betroffenen Neugeborenen auftreten, weshalb eine umgehende kardiologische Untersuchung durchgeführt werden sollte. Weitere seltene Organmanifestationen umfassen Leber- und Blutbildveränderungen, wie Anämie, Thrombozytopenie oder Leukozytopenie [1]. Bei Verdacht auf eine noch nicht diagnostizierte Kollagenose bei einer schwangeren Frau, wird ein ANA-Screening vor der 10.–12. Schwangerschaftswoche angeraten. Wenn bei der Kindesmutter Anti-SSA/Ro- oder Anti-SSB/La-Antikörper festgestellt werden, wird die fetale Echokardiographie ab der Gestationswoche 16 empfohlen. Zusätzlich soll eine Therapie mit Hydroxychloroquin 400 mg täglich über die gesamte Dauer der Schwangerschaft angedacht werden, um der Entstehung eines AV-Blockes beim Feten vorzubeugen [6, 7].

## Fazit für die Praxis

- Bei kongenitalen periorbitalen Hautveränderungen muss differenzialdiagnostisch immer ein neonataler Lupus erythematodes (NLE) in Betracht gezogen und umgehend eine serologische sowie kardiologische Untersuchung veranlasst werden.
- Bei negativer Anamnese muss zusätzlich eine umfangreiche klinische und serologische Untersuchung der Kindesmutter erfolgen.
